# Rapid, High Affinity Binding by a Fluorescein Templated Copolymer Combining Covalent, Hydrophobic, and Acid–Base Noncovalent Crosslinks

**DOI:** 10.3390/s18051330

**Published:** 2018-04-25

**Authors:** Casey J. Grenier, Anthony Timberman, Rongfang Yang, John Csoros, Alex Papantones, Leila F. Deravi, W. Rudolf Seitz

**Affiliations:** 1 Tekscan, Inc., 307 W. First St., Boston, MA 02127, USA; cgren1@gmail.com; 2 Department of Chemistry, University of New Hampshire, Durham NH 03824, USA; adt2002@wildcats.unh.edu (A.T.); rke22@wildcats.unh.edu (R.Y.); jrz56@wildcats.unh.edu (J.C.); 3 Boston Analytical, 14 Manor Parkway, Salem, NH 03079, USA; alex442@comcast.net; 4 Department of Chemistry and Chemical Biology, Northeastern University, Boston, MA 02115, USA; l.deravi@northeastern.edu

**Keywords:** templates, moleculary imprinted polymers, poly(*N*-isopropylacrylamide), noncovalent crosslinks, fluorescein, binding affinity, binding kinetics

## Abstract

A new type of biomimetic templated copolymer has been prepared by reverse addition fragmentation chain transfer polymerization (RAFT) in dioxane. The initial formulation includes the template fluorescein, *N*-isopropylacrylamide (NIPAM, 84 mol %), methacrylic acid (MAA, 5-mol %), 4-vinylpyridine (4-VP, 9 mmol %), and *N*,*N*′-methylenebis(acrylamide) (MBA, 2 mol %). PolyNIPAM is a thermosensitive polymer that comes out of aqueous solution above its lower critical solution temperature forming hydrophobic ‘crosslinks’. MAA and 4-VP interact in dioxane forming acid–base crosslinks. The excess 4-VP serves as a recognition monomer organizing around the template fluorescein to form a binding site that is held in place by the noncovalent and covalent crosslinks. The MBA is a covalent crosslinker. The RAFT agent in the resulting copolylmer was reduced to a thiol and attached to gold nanoparticles. The gold nanoparticle bound copolymer binds fluorescein completely in less than two seconds with an affinity constant greater than 10^8^ M^−1^. A reference copolymer prepared with the same monomers by the same procedure binds fluorescein much more weakly.

## 1. Introduction

Templating is widely used to introduce selective binding sites into polymers [1,2,3,4]. Recognition monomers assemble around the template creating a binding site that is held in place by crosslinks [5,6,7]. These materials, generally known as molecularly imprinted polymers, have been touted as replacements for antibodies as the recognition elements in chemical sensors, offering the advantages of stability and low cost preparation [4,8,9,10]. However, these advantages have not been realized in practice [9,11]. High levels of covalent crosslinkers are used to prepare most templated copolymers resulting in rigid materials with binding constants on the order of 10^5^ to 10^7^ M^−1^ and binding times that are often on the order of hours [12,13,14,15]. The binding rates are not acceptable for most chemical sensing applications and the binding constants are too small to measure analytes that are present at low concentrations [4,8,12,16].

Natural receptors such as antibodies and enzymes do not rely on extensive covalent crosslinking [17]. Instead, their conformations are established by predominantly noncovalent interactions including hydrogen bonding and hydrophobic interactions [18,19,20]. This suggests an alternative approach to templating that will result in a more flexible polymer that will bind more rapidly [21,22].

The possibility of preparing templated polymers with a low percentage of covalent crosslinks was demonstrated by Watanabe et al. with a copolymer of poly(*N*-isopropylacryamide) (polyNIPAM) [23]. PolyNIPAM is well known to undergo a thermal phase transition. It is soluble in water at temperatures below 32 °C and comes out of solution above that temperature [24,25]. The temperature of the phase change is known as the lower critical solution temperature (LCST) [26]. Below the LCST, hydrogen bonding between water and the amide group is strong enough to keep polyNIPAM in solution. Hydrogen bonding gets weaker with increasing temperature. Above the LCST, hydrophobic interactions are stronger than hydrogen bonding causing polyNIPAM to come out of solution [22,27,28,29,30,31,32].

Watanabe et al. demonstrated that a templated polyNIPAM copolymer prepared in dioxane with 5 mol % covalent crosslinker selectively binds to templates at temperatures above the LCST. We hypothesize that above the LCST, polyNIPAM forms noncovalent crosslinks via hydrophobic interactions, helping to hold the binding site in a conformation that selectively interacts with the template. While this study demonstrated selective binding using a lower percentage of covalent crosslinks, it did not involve either affinity constant or binding kinetic measurements [23].

Using the same approach, we showed that lightly crosslinked polyNIPAM copolymers templated with theophylline responded to concentrations as low as 1 × 10^−7^ M and did not respond to caffeine concentrations as high as 1 × 10^−3^ M, even though caffeine differs from theophylline by the presence of a single methyl group [21].

In the work reported here, we have extended the earlier work by further reducing the extent of covalent crosslinking to 2 mol % in the initial formulation and by adding ‘acid–base’ crosslinks by including both acidic and basic monomers in the formulation [33]. We chose to use fluorescein, a highly efficient fluorophore, in exploring this approach because fluorescein is easily measured at very low concentrations. Using fluorescein as the template we have measured for the first time the affinity constant and the template binding kinetics for a templated copolymer with predominantly noncovalent crosslinks and show that they greatly improve on the values typically observed with high crosslinked templated copolymers. Our results suggest that this strategy may be more successful in preparing recognition elements with the required properties for chemical sensors.

## 2. Experimental

Reagents: Methacrylic acid (MAA) from Sigma-Aldrich (St. Louis, MO, USA) was vacuum distilled, then passed through columns of basic alumina and inhibitor remover to remove inhibitor. 4-Vinylpyridine from Sigma-Aldrich was vacuum distilled, then passed thought a column of basic alumina and a column of inhibitor remover to remove inhibitor.

*N*-Isopropylacrylamide at 99% purity (NIPAM) from TCI was recrystallized from hexane three times to remove inhibitor. *N*,*N*′-methylenebis(acrylamide) at 99% purity from Sigma-Aldrich was recrystallized from methanol three times. The following chemicals were obtained from Sigma-Aldrich and used as received: 2,2 azobisisobutyronitrile (AIBN); the free acid form of fluorescein; inhibitor removal beads; 1,4-dioxane, 99.8% anhydrous; methanol ACS grade: hexane, ACS grade; and 2-(dodecylthiocarbonothioylthio)2-methylpropanoic acid 97% (DDMAT).

Sigma-Aldrich was also the source for 20 nm diameter gold nanoparticles stabilized in 0.1 mM phosphate buffered saline (7.2 × 10^11^ particles/mL).

Apparatus: Dynamic light scattering (DLS) was used to determine particle size on a Malvern Zetasizer Nano NS. NMR spectra were measured on a Varian 400 MHz Mercury Liquid State NMR. Gel permeation chromatography was performed on an Agilent 1260 GPC with a Polymer Lab aqueous SEC column. Fluorescence was measured with a Cary Eclipse fluorescence spectrophotometer with a Peltier thermostatted single cell holder. A 0.70 mL cell was used for the fluorescence measurements.

A Branson model 1210 sonicator was used for reagent dissolution, nanoparticle mixing, and sonication. A Buchi RE111 Rotovapor was used to evaporate solvents. An Eppendorf centrifuge 5415D (13,200 rpm) was used to separate polymer coated gold nanoparticles from solution. A Labconco FreeZone 1 Lite Bench Top Freeze Dry System coupled with a stainless-steel tower with four ports and airable glass containers was used to remove water from the polymer yielding a powder. A Synthware Four Port Schlenk line coupled with an Alcatel oil pump was used to perform freeze–pump–thaw degassing and vacuum distillation. Dialysis tubing, Sigma-Aldrich MWCO 10,000 to 12,000, was used to remove the template from polymer.

Procedures: Polymers were prepared by reversible addition-fragmentation chain transfer (RAFT), a form of living polymerization. The polymerization was carried out using 42.5 mmol NIPAM, 4.5 mmol 4-VP, 2.5 mmol MMA and 1 mmol MBA in 500 mL of dioxane. The polymerization solution was degassed by freeze-pump-thawing three times and then backfilled with nitrogen. DDMAT was used as the chain transfer agent for RAFT and AIBN was used as the initiator. The monomer:initiator:RAFT agent ratio was 1000:1:1. The solution was polymerized for 24 h at 70 °C. Two polymers were prepared for this study. One contained 0.5 mmol fluorescein. This polymer is designated TMP (for template). The other polymer was prepared identically except that fluorescein was omitted. This polymer is designated REF (for reference).

After polymerization, unreacted monomers and template—including any template trapped in the polymer matrix—were removed by dialysis. The solution was initially placed in a dialysis bag and dialyzed against with deionized water four times at 2 to 4 h intervals. After this the dialysis solutions was rotated between 0.10 M NaOH and an aqueous solution with 30% (*v*/*v*) methanol and 5% (*v*/*v*) acetic acid at 8 to 12 h intervals for a week. After this we dialyzed for a day in deionized water with one hour intervals. The next day we used 50% (*v*/*v*) methanol as the external solution. The pattern was then repeated. After three to four weeks, the solid was lyophilized. It was then redissolved in water and the procedure was repeated. Altogether, it took six months to remove all the fluorescein from the templated copolymer. When polymers are dissolved in water the polymer chains are tangled. This will block access to some of the binding sites. Hydrophobic interactions between isopropyl groups will retard the rate at which pNIPAM copolymers untangles. We believe that chain tangling is blocking access to binding sites, resulting in slow removal of template from the copolymer. We changed dialysis media with the idea that this might cause the chains to rearrange to explose new binding sites. We have since learned that template can be removed in two to three weeks if we include ca. 20% (*v*/*v*) tetrahydrofuran in the solutions used for dialysis. Because, as shown below, template binding is rapid, we would expect template removal to more rapid if the copolymer chains were not tangled.

Both TMP and REF were attached to gold nanoparticles by a published procedure [34]. The polymers prepared by RAFT have the RAFT agent at one terminus. This is reduced to a thiol by sodium borohydride and then exposed to gold nanoparticles.

Binding affinity was measured using polyacrylic or high-density polyethylene equilibrium dialysis cells in a water bath for temperature control. Spectra/Por 3 flat dialysis sheet, MWCO 3.5 kD, separated the two cell compartments. A fluorescein solution was added to one side of the cell and copolymer solution was added to the other side. After equilibrating for at least 48 h, the concentration of fluorescein remaining on the fluorescein side of the equilibrium dialysis cell was measured by fluorescence. This value was used to calculate the distribution ratio, i.e., the concentration on the polymer side of the cell divided by the concentration on the side of the cell without polymer. Equilibrium dialysis experiments were conducted at room temperature.

All binding experiments were conducted in a 0.10 M phosphate buffer that controlled the pH at 7.2.

## 3. Results and Discussion

Polymer Characteristics: Both copolymers, TMP and REF, form a clear solution in water at room temperature. In this respect, they differ from typical template copolymers that involve a high degree of crosslinking that causes them to be solids.

The molar masses of TMP and REF were determined by gel permeation chromatography to be 13,800 and 13,600 g/mol, respectively. The theoretical chain length for polymers prepared by RAFT is determined by the ratio of monomer to RAFT agent in the initial formulation. It would be over 100,000 g/mol. Thus, the low molar mass observed by GPC suggests that the polymerization is not close to completion.

The polydispersity indices were 2.18 and 2.15 respectively. This is much higher than typical polydispersity indices for polymers prepared by RAFT. It means we have polymer chains that vary in the number of monomer units per chain. We attribute this to interchain crosslinking by methylenebisacrylamide. This will connect two chains, leading to a chain with more monomer units.

**Figure 1:**
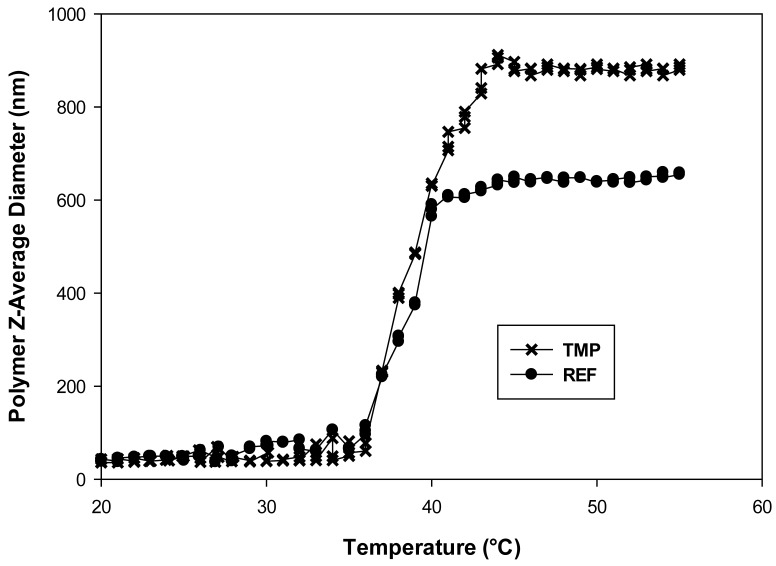
DLS of the TMP and REF at 1 g/L in DI Water (filtered twice with a 0.45 PET syringe filter) changing polymer size) versus temperature. x-x-x is for TMP, •-•-• is REF.

Dynamic light scattering data is shown in Figure 1 for TMP and REF. These data show particle size vs. temperature for copolymers prepared in the presence and absence of fluorescein. The polymer prepared with fluorescein was tested before template removal by dialysis. Based on binding capacity measurements presented later in this manuscript, the templated polymer contains 0.013 g of fluorescein per gram of polymer. Both polymers show the characteristic polyNIPAM phase transition, aggregating at elevated temperatures. However, the LCST is shifted to higher temperatures relative to pure polyNIPAM which has an LCST of 32 °C. We attribute this to the presence of hydrophilic comonomers, particularly MAA. If we take the LCST as the point at which the copolymer size is half way between its values at low and high temperatures, then the LCST is 39 °C for TMP and 37 °C for REF.

Added fluorescein did not affect the DLS data for the REF polymer. This is expected if the fluorescein does not interact with the polymer. These experiments show that the presence of a template during polymerization affects the degree to which the copolymers aggregate above the LCST.

Interestingly, the templated copolymer forms much larger aggregates than the untemplated copolymer, suggesting that the two copolymers have different conformations.

NMR spectra of the copolymers in D_2_O show peaks due to aromatic protons of 4-VP that allow us to calculate actual percentages of 4-VP in the copolymer. They show that the actual mole percentages of 4-VP are 7% for TMP and 4% for REF. The presence of the template fluorescein during polymerization appears to promote the incorporation of 4-VP into the copolymer. Even though TMP and REF were prepared using the same initial monomer concentrations, the resulting copolymers do not have the same distribution of monomers. The fact that the LCST for TMP is higher than the LCST for REF suggests that TMP has higher mol-percentages of hydrophilic monomers.

A separate NMR experiment was performed to demonstrate that vinyl pyridine and methacrylic acid interact in dioxane. Spectra were measured for 6.7 M methacrylic acid in deuterated dioxane in the presence and absence of 6.7 M vinyl pyridine. When methacrylic acid is by itself the carboxylic acid proton is observed as a band close to δ = 9. In the presence of vinyl pyridine, this band shifts to 11. These data confirm our hypothesis that the acid and base monomers interact with each other, a process that will from a crosslink.

Binding Constant Measurements: Binding constants were measured both by equilibrium dialysis and by attaching the polymer to gold nanoparticles that could be removed from suspension by centrifugation.

Table 1 shows the distribution ratio as a function of copolymer concentration measured by equilibrium dialysis at the LCST. The results are remarkable. The data for TMP show that the extent of binding increased when the concentration of copolymer in the equilibrium dialysis cell decreased. When a copolymer goes into solution, the individual chains are entangled with each other. This can block access to binding sites, resulting in weaker binding. Hydrophobic interactions between the isopropyl groups retard the rate of untangling. As the concentration is reduced, the chains will be less entangled and there will be greater access to binding sites. The increase in binding with decreasing concentration suggests that this is occurring.

Table 1 includes data for REF. The distribution ratio for REF approaches 1.00 as the polymer concentration decreases. All experiments had 100 nM of fluorescein present starting on the template side of the equilibrium dialysis block. This experiment gives us the extent of nonspecific binding for an untemplated copolymer.

The data in Table 1 are very promising in that they show by far the best results at low copolymer concentrations. These are the concentrations that are preferred for most applications.

The data in Table 1 do not allow us to calculate an affinity constant because we do not know the total number of template binding sites on the polymer. 

**Figure 2:**
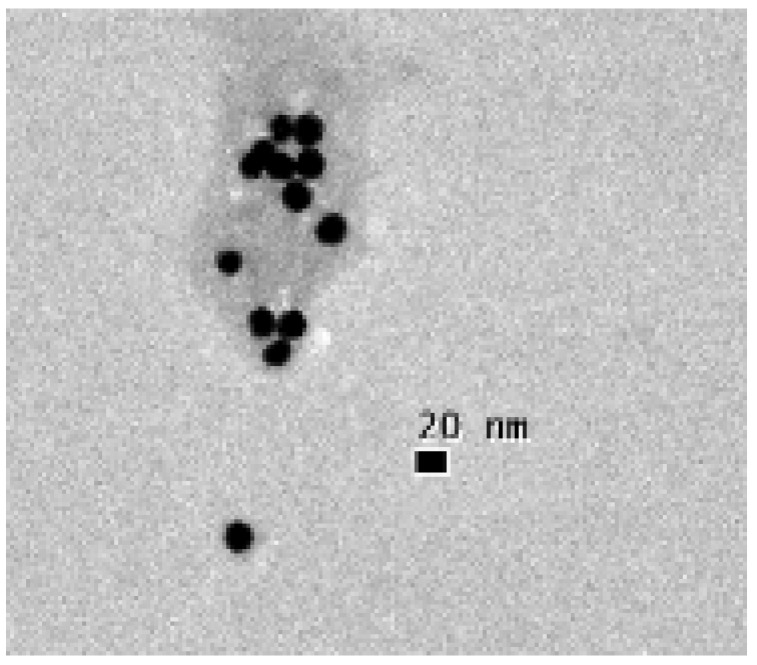
Transmission electron microscopy images of AuNP stabilized with TEM polymer. The scale bare is equal to 20 nm.

We performed a different experiment to measure the affinity constant and the binding capacity. Copolymers prepared by the RAFT include the RAFT agent at one terminus. This was reduced to a thiol using sodium borohydride. 100 µL of a 0.0355 g/L copolymer solution were combined with 900 µL of a gold nanoparticle (AuNP) suspension, producing a suspension with 3.55 µg copolymer on 122.0 µg of AuNP [34,35]. Some of this suspension was placed on a grid and dried for transmission electron microscopy. Figure 2 shows micrographs for this sample, confirming that polymer (light gray) is attached to the AuNP (black spheres).

**Figure 3:**
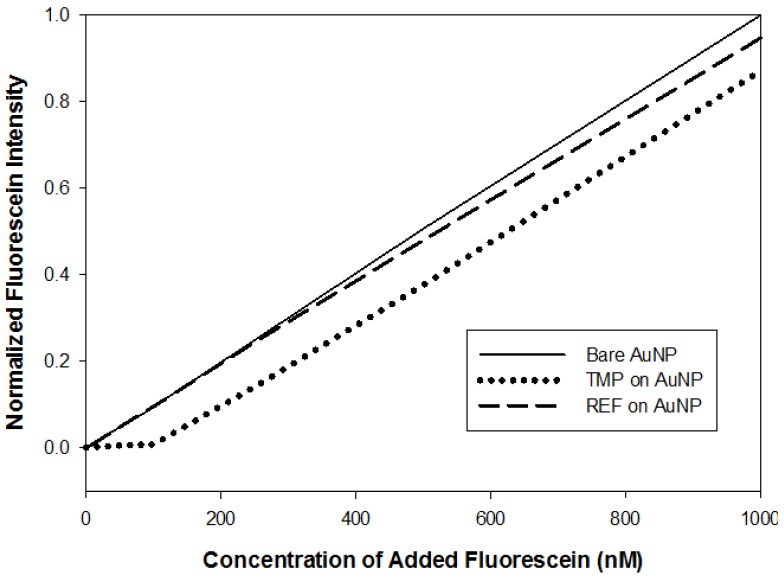
TMP and REF (0.0355 g/L) stabilized on to multiple 20 nm AuNP samples then added to varying concentrations of fluorescein (10–1000 nM) aliquots. The various solutions were all heated to the corresponding LCST and then spun down, the supernatant was removed and analyzed for remaining unbound fluorescein.

This sample was titrated with fluorescein at the LCST. After each addition of fluorescein, the suspension was allowed to equilibrate before the AuNP were removed by centrifugation. The concentration of fluorescein in the supernatant was then measured by fluorescence. Figure 3 shows the results of this experiment performed with TMP and REF on AuNP and with bare AuNP. On bare AuNP, we see the expected linear plot of measured fluorescence intensity at 512 nm vs. fluorescein concentration. The slope for REF bound to AuNP, indicating that there is some nonspecific binding of fluorescein to the untemplated copolymer. This is consistent with the distribution ratio slightly greater than 1 that we observe for the equilibrium dialysis experiments with REF (Table 1).

The presence of TMP drastically reduces the concentration of fluorescein in the supernatant for low concentrations. However, as the fluorescein levels are increased, the fluorescence intensities increase and the slope of the fluorescence vs. fluorescein concentration curve equals the slope of the calibration curve with bare AuNP. 

**Figure 4:**
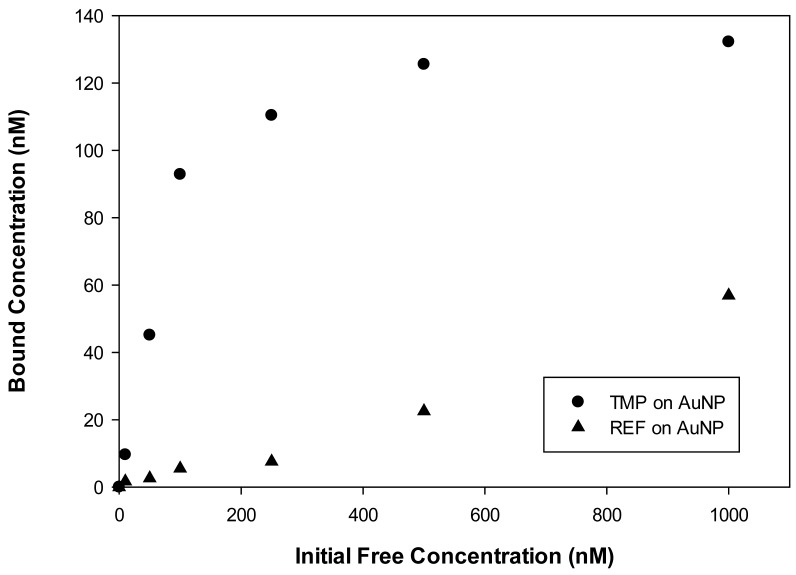
Bound fluorescein to the TMP/REF stabilized AuNP versus initial fluorescein present before it was introduced and heated to the LCST of TMP or REF. Concentration of the bound fluorescein is calculated from the data from Figure 3.

Since we know both the total fluorescein concentration and the fluorescein concentration remaining in the supernatant we can calculate the concentration of fluorescein bound to copolymer at equilibrium. These values are plotted vs. added fluorescein for TMP coated AuNP in Figure 4. This plot shows that the bound concentration reaches a maximum value with added fluorescein. Further added fluorescein does not cause a further increase in bound fluorescein. The maximum value that we observe is the binding capacity. 

At low fluorescein concentration, only some of the binding sites are occupied. The difference between the binding capacity and the actually number of occupied binding sites allows us to calculate the number of unoccupied binding. From these data, we can calculate the binding affinity, which is equal to number of occupied binding sites divided by the number of unoccupied binding sites times the solution concentration of fluorescein.

The binding capacity is 0.013 g fluorescein per gram of polymer. On a mole basis we have just over half a mole of fluorescein per mole of copolymer. Approximately half the polymer molecules have binding sites for fluorescein.

We also calculate 13.5 mole of the recognition monomer, 4-VP, per mole of fluorescein. If each fluorescein binding site has 4 moles of 4-VP, this leaves 9.5 moles of 4-VP that are not involved in binding and available to interact with methyacrylic acid to form acid–base crosslinks.

Since we know the number of binding sites, we can calculate the affinity constant

$$K_{\mathrm{affinity}}=\frac{\mathrm{Occupied}\;\mathrm{binding}\;\mathrm{sites}}{\mathrm{Open}\;\mathrm{binding}\;\mathrm{sites}\;\times \left[\mathrm{Fluorescein}\right]}$$

We can calculate an affinity constant at each value of added fluorescein. The highest calculated value is at 100 nM added fluorescein and comes out to be 2.3 × 10^8^ M^−1^.

**Figure 5:**
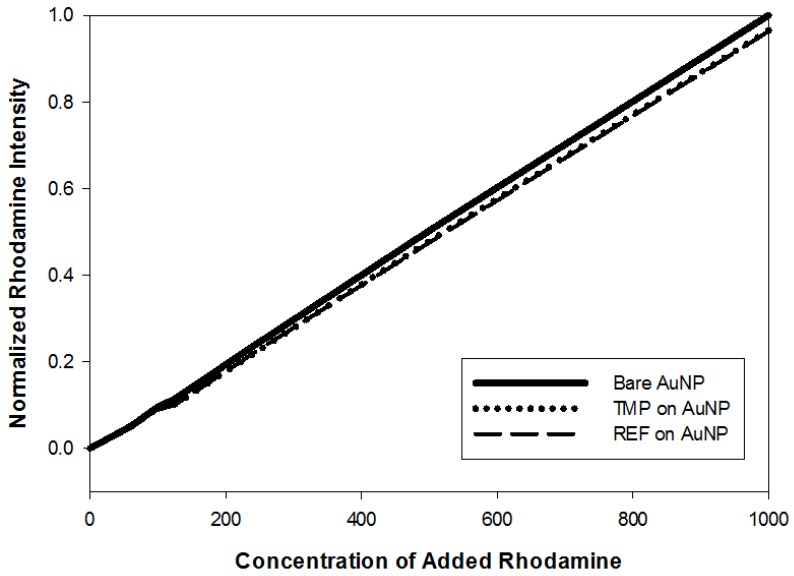
TMP and REF (0.035 g/L) stabilized on to multiple 20 nm AuNP samples then added to varying concentrations of rhodamine-B (10–1000 nM) aliquots. The various solutions were all heated to the corresponding LCST then spun down and the supernatant was removed and analyzed for remaining unbound rhodamine-B.

Selectivity: We evaluated selectivity by measuring the response to rhodamine B, a fluorescent dye that is similar in shape to fluorescein but has different functional groups. Figure 5 shows the intensity of rhodamine fluorescence as a function of concentration for bare AuNPs and for AuNPs coated with TMP and REF. The presence of polymer on the gold nanoparticles lowers the slope of this plot, indicating a low degree of binding. The slope of the intensity vs. rhodamine B concentration curve is the same for both the templated and untemplated copolymer on gold, indicating that templating with fluorescein does not affect how the copolymer binds to rhodamine B. 

**Figure 6:**
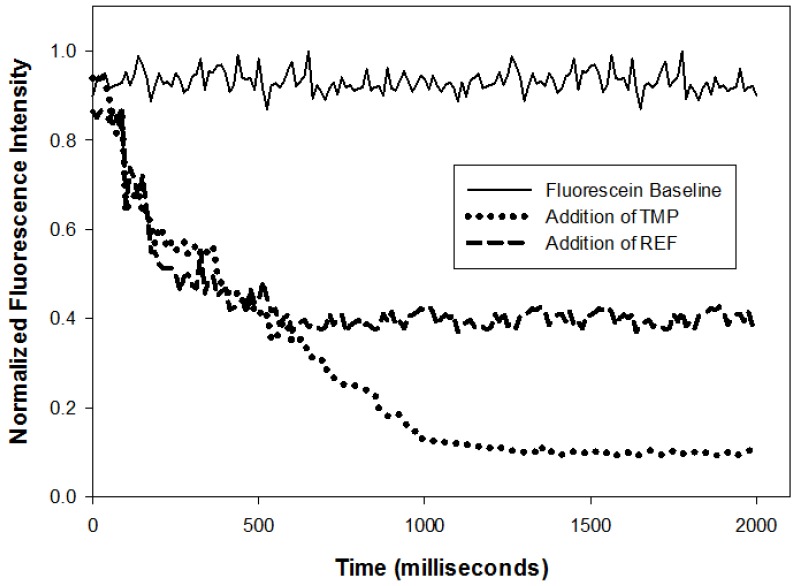
Initial amount of 100 nM fluorescein in DI water was taken for a fluorescence baseline. Equal parts of fluorescein (100 nM) and TMP polymer solution (0.0355 g/L) were mixed together and normalized fluorescence intensity was measured over time (milliseconds). The same experiment was repeated with equal parts fluorescein (100 nM) and REF polymer solution (0.0355 g/L).

Binding Kinetics: Because our copolymer has fewer crosslinks and more flexibility than typical templated polymers, we expected it to react more rapidly. This was tested by combining equal volumes of a fluorescein solution and a suspension of copolymer coated AuNPs in a stirred fluorescent cuvette and measuring intensity vs. time at 40 °C. The results of this experiment are summarized in Figure 6. When fluorescein solution is mixed with deionized water or with REF coated AuNPs, the observed intensity decreases by ca. 50% as expected for dilution. When fluorescein solution is combined with TMP coated particles, the intensity decrease is larger and it takes longer for the intensity to reach a constant value. We attribute the extra decrease in intensity to concentration quenching because binding will bring fluorescein molecules into close proximity.

While this experiment is too crude to permit an accurate rate constant measurement, it does show that binding is complete in 1.5 s, a considerable improvement on binding rates reported in the literature (2, 6, 10, 12). There are two reasons why our material responds more rapidly than other templated copolymers. One is the increased flexibility because our crosslinks are noncovalent and dynamic. The other is that high degrees of covalent crosslinking inevitably produce a bulk phase rather than a copolymer that is soluble in water. In these materials, the rate of response depends on how rapidly the template diffuses through the bulk phase. 

## 4. Conclusions

The data presented here show that a templated copolymer with predominantly noncovalent crosslinks binds rapidly and selectively with larger affinity constants than structurally rigid conventional templated copolymers with much higher levels of covalent crosslinking. Further work needs to be done to characterize this type of templated copolymer, to establish the nature of the crosslinking and to further evaluate selectivity. Nevertheless, the approach presented here, which mimics nature, suggests a useful new strategy for preparing high affinity templated copolymers with rapid binding kinetics.

## Figures and Tables

**Table 1:** Distribution ratios of various concentrations of polymer TMP and REF measured at their respective LCST values. An initial concentration of 100 nM of fluorescein was used for all concentrations of polymer.

Polymer	Concentration (g/L)	Distribution Ratio (at LCST)
*TMP*	10	3.67
*TMP*	1	4.10
*TMP*	0.1	4.57
*TMP*	0.036	9.89
*REF*	10	1.73
*REF*	1	1.34
*REF*	0.1	1.28
*REF*	0.036	1.10
